# Maternal Undernutrition and Low Birth Weight in a Tertiary Hospital in Sudan: A Cross-Sectional Study

**DOI:** 10.3389/fped.2022.927518

**Published:** 2022-06-21

**Authors:** Jalal A. Bilal, Duria A. Rayis, Ashwaq AlEed, Abdullah Al-Nafeesah, Ishag Adam

**Affiliations:** ^1^Department of Pediatrics, College of Medicine, Shaqra University, Shaqra, Saudi Arabia; ^2^Department of Obstetrics and Gynaecology, Faculty of Medicine, University of Khartoum, Khartoum, Sudan; ^3^Department of Pediatrics, Unaizah College of Medicine and Medical Sciences, Qassim University, Unaizah, Saudi Arabia; ^4^Department of Pediatrics, College of Medicine, Qassim University, Buraydah, Saudi Arabia; ^5^Department of Obstetrics and Gynecology, Unaizah College of Medicine and Medical Sciences, Qassim University, Unaizah, Saudi Arabia

**Keywords:** undernutrition, pregnant women, low birth weight, Sudan, cross sectional study

## Abstract

**Background:**

The World Health Organization set a Global Nutrition Target of a 30% reduction in LBW by 2025. Maternal malnutrition/undernutrition is among the most important modifiable risk factors for impaired fetal growth. This study investigates the effect of maternal undernutrition on LBW in Sudan.

**Methods:**

A cross-sectional study was conducted at Saad Abuelela Hospital in Khartoum, Sudan, from May to October 2020. The sociodemographic and obstetric data of the women were gathered *via* questionnaire, and their mid-upper arm circumference (MUAC) was measured. Maternal undernutrition was defined as a MUAC of <23 cm.

**Results:**

In total, 1,505 pairs of pregnant women and their newborns were enrolled in the study. The medians [interquartile (IQR)] of the age, parity, and gestational age were 27.0 (9.0) years, 1.0 (3.0), and 38.0 (2.0) weeks, respectively. The median (IQR) of the birth weight was 3,028.0 (690.0) g. Of the 1,505 participants, 182 (12.1%) delivered LBW infants. Multivariate logistic regression showed that MUAC [adjusted odds ratio (AOR) = 0.91, 95% confidence interval (CI) = 0.87–0.96] and gestational age (AOR = 0.79, 95% CI = 0.73–0.85) were negatively associated with LBW. The level of antenatal care <2 visits (AOR = 2.10, 95% CI = 1.30–3.57) was associated with LBW. Women with undernutrition were at a higher risk of delivering LBW infants (AOR = 1.66, 95% CI = 1.09–2.53).

**Conclusion:**

LBW is a health problem in Sudan, and women with undernutrition were at a higher risk of delivering LBW infants.

## Introduction

It has been estimated that over 20 million deliveries have resulted in LBW infants (LBW; <2,500 g) annually; the vast majority of these LBW deliveries are in low- and middle-income countries (1). Several factors, such as infections, low education level, low income, and occupation, are associated with LBW (2–4). It has been found that about 3.6 million infants die (mainly in southern Asia and sub-Saharan Africa) during the neonatal period (1). The World Health Organization (WHO) set a Global Nutrition Target of a 30% reduction in LBW by 2025 (1). Maternal nutrition affects the growth of the fetus as well as birth and neonatal outcomes (5–8). Reports indicate that more than one-third of child deaths are caused by maternal and child undernutrition (9). Maternal malnutrition/undernutrition is among the most important modifiable risk factors for impaired fetal growth (9). Nutrition plays a fundamental role in health of pregnant women and the growth of fetuses. Poor maternal nutrition can lead to an increased risk of stillbirth, an increased risk of neonatal morbidity, death, and permanent deficits in growth and neurocognitive development (10).

Maternal undernutrition is a problem in developing countries (11). The prevalence of undernutrition among pregnant African women was 23.5% (12). It has been estimated that up to 20% of African women of reproductive age are undernourished (9, 13, 14).

Investigating the association between maternal undernutrition and LBW is vital for evidence-based interventions to reduce the burden of LBW. Several studies have assessed the association between birth weight/LBW and maternal undernutrition in African countries (15–19). LBW is a health problem in Sudan (20, 21). It has been reported that 12.5% of pregnant Sudanese women in Khartoum are undernourished (22). To the best of our knowledge, there are no evidence-based publications on the association between maternal undernutrition and LBW in Sudan. As such, this study was conducted to investigate the effect of maternal undernutrition on LBW in Sudanese women.

## Materials and Methods

This cross-sectional study was conducted at Saad Abuelela Hospital in Khartoum, Sudan, from May to October 2020.

### Inclusion Criteria

Women with a single and alive newborn.

### Exclusion Criteria

**FIGURE 1:**
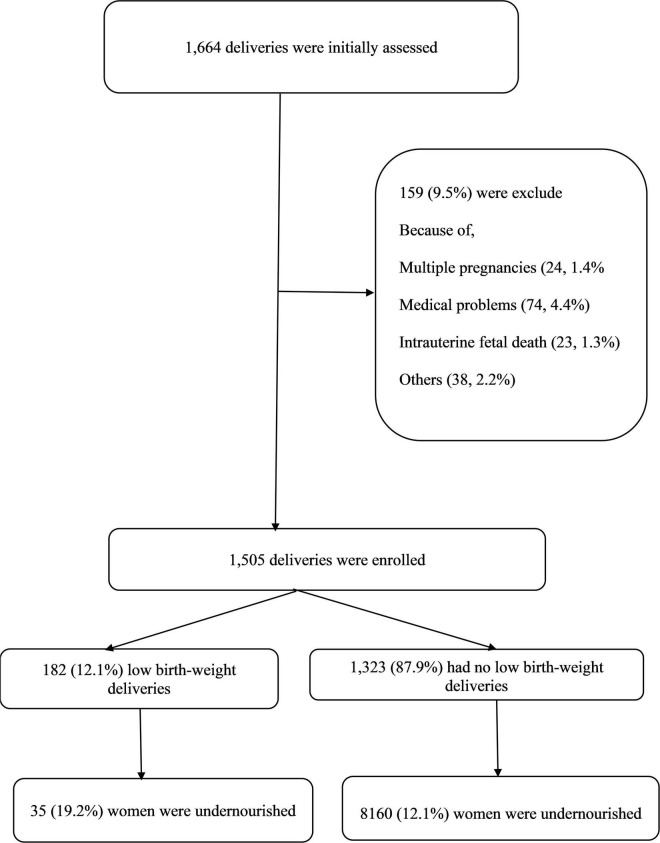
The flow chart for the study.

Women with multiple pregnancies, intra-uterine fetal death, delivering a baby with one or more congenital anomalies, and women with diseases known to influence the birth weight such as thyroid disease, diabetes mellitus, hypertension, antepartum hemorrhage, or any other chronic disease (Figure 1) were excluded.

After signing informed consent, data were collected by trained medical officers who graduated from medical schools. The socio-demographic and obstetric data were gathered and recorded face-to-face using a structured questionnaire about obstetric history (age, parity, gestational age, antenatal attendance, education level, miscarriage history, and employment). Gestational age was calculated using a combination of the dates of the last menstrual period and early pregnancy ultrasound.

Newborn weights were recorded within 1 h of delivery. The mother’s mid-upper arm circumference (MUAC) was measured after delivery using a flexible non-stretchable standard tape measure. The circumference was measured at the mid-point between the tip of the acromion process of the scapula and the olecranon process of the ulna. Measurements were taken on the right arm to the nearest 0.1 cm (23). Maternal undernutrition was defined as a MUAC of <23 cm. Hemoglobin was measured (before delivery) using an automated hematology analyzer according to the manufacturer’s instructions (Sysmex, KX-21, Japan). A Salter scale (which was checked for accuracy daily) was used to weigh the newborns immediately (by the staff) after birth to the nearest 10 g. The gender of each newborn was recorded.

### Sample Size Determination

A sample size calculation for a cross-sectional study was applied and was estimated as 1,505 women using the recent prevalence (14.5%) of LBW in the study area (20). Thus, we assumed that the ratio of women with LBW to the women with no LBW was 1:6. Depending on our findings, we expect that 20.0% of the women who had LBW had undernutrition, and 12.5% of the women who had no LBW had undernutrition (22). This sample had a type I error of 5% and adequate power (80% power; β = 0.2).

### Statistics

Data were entered into the Statistical Package for Social Sciences (SPSS) version 22 software (SPSS Inc.) for analysis. Continuous data (including MUAC) were checked for normality and were not normally disturbed. Therefore, the median [interquartile (IQR) range] was used to express their values. Categorized data were presented as frequencies and proportions. Multicollinearity (variance inflation factor, <4) was checked for but not detected. Univariate analyses were performed with LBW as the dependent variable and clinical obstetrics data [age, parity, employment, education level, antenatal care (ANC), MUAC, hemoglobin, gestational age, and newborn gender] as the independent variables. MUAC and undernutrition were entered one by one in each model. Variables with a *p* < 0.20 univariate analysis were entered to build the multivariable logistic regression models, and backward-stepwise regression was used for adjustment. Odds ratios (ORs) with 95% confidence intervals (CIs) were calculated. A two-sided *p* < 0.05 was considered statistically significant.

## Results

### Basic Characteristics of the Participants

A total of 1,505 pairs of pregnant women and their newborns were enrolled in the study. The median (IQR) of the age, parity, and gestational age was 27.0 (9.0) years, 1.0 (3.0), and 38.0 (2.0) weeks, respectively. Half of these women were primipara (170, 50.1%). The vast majority of these women were housewives (1,331, 88.8%), and over half (836, 55.5%) had an education level ≥ secondary school. In total, 106 (7.0%) attended ≥ three antenatal visits, and 328 (21.8%) of the studied women had a history of miscarriage. Three hundred and twelve (20.7%) women had a cesarean delivery.

The median (IQR) of the birth weight was 3,028.0 (690.0) g. Of 1,505 participants, 182 (12.1%) delivered LBW infants.

The MUAC, gestational age, ANC, and education levels were significantly lower in women who delivered LBW infants. No significant difference was found in age, parity, hemoglobin levels, residence, employment, miscarriage history, and newborn gender between the two groups (Table 1).

**TABLE 1:** Univariate analysis of the factors associated with low birth weight in Khartoum, Sudan, 2020.

Variables	Low birth weight (182)	No low birth weight (1,323)			
	Median (interquartile range)		OR	95% CI	*p*-value
Age (years)	26.5 (11.0)	27.0 (9.0)	0.98	0.95–1.01	0.197
Parity	1 (3.0)	1 (3.0)	0.99	091–1.05	0.987
Mid-upper arm circumference, cm*	25.2 (5.0)	26.0 (5.0)	0.91	0.88–0.96	<0.001
Hemoglobin level (g/dl)	10.7 (1.9)	10.5 (1.6)	1.02	0.91–1.56	0.637
Gestational age, weeks	38.3 (3.0)	38.3 (3.0)	0.79	0.73–0.85	<0.001
	**Number (percentage)**				
**Education level**					
Secondary or higher	85 (46.7)	751 (65.8)	Reference		
Primary or lower	97 (53.3)	572 (43.2)	1.49	1.09–2.04	0.001
**Residence**					
Urban	93 (51.1)	722 (56.6)	Reference		
Rural	89 (48.9)	601 (45.4)	1.15	0.84–1.56	0.378
**Employment**					
Housewives	163 (89.6)	1,168 (88.3)	Reference		
Non-housewives	19 (10.4)	155 (11.7)	0.87	0.53–1.45	0.614
Antenatal care			1.16	0.68–1.88	0.614
≥2 visits	154 (84.6)	1,245 (91.4)	Reference		
<2 visits	28 (15.4)	78 (5.9)	2.90	1.82–4.61	<0.001
**History of miscarriage**					
No	141 (77.5)	1,036 (78.3%)	Reference		
Yes	41 (22.5%)	287 (21.7%)	1.05	0.72–1.52	0.789
**Undernutrition** *					
No	147 (80.8)	1,163 (87.9)	Reference		
Yes	35 (19.2)	160 (12.1)	1.73	1.51–2.59	0.008
**Gender**					
Female	101 (55.5)	660 (49.9)	Reference		
Male	81 (44.5)	663 (50.1)	0.80	0.58–1.09	0.163

CI, confidence interval; OR, odds ratio. *stand for median.

The median (IQR) of MUAC was significantly lower in women who gave birth to LBW infants [10.0 (6.5) ng/mL vs. 18.3 (22.1) ng/mL]. In total, 35/182 (19.2%) women with LBW infant and 160/1,323 (12.1%, *p* = 0.010) women who did not deliver a LBW infant had undernutrition.

Multivariate logistic regression showed that MUAC [adjusted odds ratio (AOR) = 0.91, 95% CI = 0.87–0.96] and gestational age (AOR = 0.79, 95% CI = 0.73–0.85) were negatively associated with LBW. A level of antenatal care <2 visits (AOR = 2.10, 95% CI = 1.30–3.57) was associated with LBW. Women with undernutrition were at a higher risk of delivering LBW infants (AOR = 1.66, 95% CI = 1.09–2.53), Table 2.

**TABLE 2:** Logistic regression analysis of the factors associated with low birth weight in Khartoum, Sudan, 2020.

Variables	Adjusted values		
	OR	95% CI	*p*-value
Age, years	1.01	0.97–1.04	0.536
Mid-upper arm circumference, cm**	0.91	0.87–0.96	<0.001
Gestational age, weeks	0.79	0.73–0.85	<0.001
**Education level**			
Secondary or higher	Reference		
Primary or lower	1.40	0.97–2.02	0.067
**Antenatal care**			
≥2 visits	Reference		
<2 visits	2.10	1.30–3.57	0.003
Undernutrition*			
No	Reference		
Yes	1.66	1.09–2.53	0.018
**Gender**			
Female	Reference		
Male	1.37	0.98–1.90	0.059

*CI, confidence interval; OR, odds ratio.*

*These were entered one by one (one per model).

**Adjusted for age and education.

## Discussion

In this study, 12% of deliveries resulted in a LBW infant. The prevalence of LBW in our study is comparable with the LBW prevalence (14.3%) which was previously reported in the same hospital (20) and in different hospitals in neighboring Ethiopia (24–26). The prevalence of LBW in our study is lower than the LBW prevalence (21.6%) reported at the Debre Markos Hospital (Ethiopia) (27). However, the prevalence of LBW in the current study is much higher than the LBW prevalence in Ghana (9.7%) (28) and in Nigeria (7.3%) (29). Notably, the prevalence of LBW in our study is lower than the pooled LBW prevalence in Sub-Saharan Africa (9.76%) recently reported in a meta-analysis (30). The LBW prevalence difference between the current study and a later one can be explained by the difference in study design and the sociodemographic characteristics of different settings. It is worth mentioning that a high LBW prevalence indicates a difficulty in achieving the World Health Assembly’s (WHA’s) target of reducing the LBW prevalence to ≤10.5% by 2025 (31).

In the current study, pregnant women who had attended less than two ANC visits were at a 2.10 higher risk of delivering an LBW newborn. This is consistent with the previous studies conducted in Sudan (32), Ethiopia (33), Kenya, Zimbabwe (34), and Tanzania (35). Moreover, in their meta-analysis, Tessema et al. reported that ANC visits were associated with reduced LBW occurrence (30). This can be explained by the opportunity for ANC to access various preventive measures (nutritional counseling and health provisions, such as iron supplements) and screen for any possible problems that might lead to LBW.

In the current study, MUAC (AOR = 0.91) was negatively associated with LBW. Women with undernutrition were at a higher risk of delivering LBW (AOR = 1.66) newborns. Several previous studies have shown that maternal undernutrition is associated with LBW (15–17). In their 2017 meta-analysis of 4,633 participants from 13 studies, Cates et al. reported that the risk of delivering a baby with LBW was associated with low MUAC (relative risk = 1.60) (18). In 2011, a meta-analysis by Han et al. of 78 studies involving 1,025,794 women found that underweight women were at an increased risk of having LBW infants (19).

Our finding of an association between gestational age and LBW is in agreement with the results from neighboring Ethiopia (15).

We reported no association between maternal age, residence, newborn gender, maternal Hb and delivering LBW infants. Previous studies have shown that maternal Hb (17), maternal age, rural residence, and female gender were significantly associated with LBW (15, 33).

The other assessment tools for the micronutrient deficiencies during pregnancy are costly and technically difficult. Anthropometrics measurements, such as MUAC, are useful in assessing the malnutrition state. Unlike body-mass index, MUAC does not change during pregnancy; therefore, it seems to be the best tool for assessing the nutritional status during pregnancy (36, 37).

### Strength and Limitations of the Study

The present study has some strengths including its large sample and taking newborn weight within 1 h of delivery. However, our study has limitations such as it was a hospital-based study, which might not reflect what was going on in the community. The study was conducted at a single hospital, the finding might not be generalizable to the entire birth cohort in the area. The cross-sectional nature of the study makes it difficult to draw inferences about the cause–effect relations among study variables. The number of ANC visits was taken from verbal response of respondents. There might be recall bias as respondents had to remember their ANC visits. In addition, important variables like physical activity and dietary diversity were not assessed.

## Conclusion

LBW is a health problem in Sudan. Women with undernutrition are at a higher risk of delivering LBW infants.

## Data Availability Statement

The raw data supporting the conclusions of this article will be made available by the authors, without undue reservation.

## Ethics Statement

The studies involving human participants were reviewed and approved by the Research and Ethical Committee of the Department of Obstetrics and Gynecology, Faculty of Medicine, University of Khartoum, Sudan (# 2020, 04). Informed consent was given by the participants. Written informed consent to participate in this study was provided by the participants’ legal guardian/next of kin.

## Author Contributions

JB designed the research, analyzed the data, and interpreted the results. AA-N and AA designed the research and wrote the manuscript. DR conducted the research, analyzed the data, and wrote the manuscript. IA designed the research, conducted the research, and analyzed the data. All authors read and approved the final manuscript.

## Conflict of Interest

The authors declare that the research was conducted in the absence of any commercial or financial relationships that could be construed as a potential conflict of interest.

## Publisher’s Note

All claims expressed in this article are solely those of the authors and do not necessarily represent those of their affiliated organizations, or those of the publisher, the editors and the reviewers. Any product that may be evaluated in this article, or claim that may be made by its manufacturer, is not guaranteed or endorsed by the publisher.
